# Cross-sectional study on the relationship between the Mediterranean Diet Score and blood lipids

**DOI:** 10.1186/1475-2891-13-88

**Published:** 2014-09-04

**Authors:** Evelien Mertens, Patrick Mullie, Benedicte Deforche, Johan Lefevre, Ruben Charlier, Inge Huybrechts, Peter Clarys

**Affiliations:** Department of Human Biometrics and Biomechanics, Faculty of Physical Education and Physiotherapy, Vrije Universiteit Brussel, Pleinlaan 2, 1050 Brussels, Belgium; Erasmus University College, Laerbeeklaan 121, 1090 Brussels, Belgium; International Prevention Research Institute (iPRI), 15 chemin du Saquin, Ecully, Lyon, France; Department of Kinesiology, Faculty of Kinesiology and Rehabilitation Sciences, KU Leuven, Tervuursevest 101, 3001 Leuven, Belgium; Department of public health, Ghent University, De Pintelaan 185, 9000 Ghent, Belgium; International Agency for Research on Cancer (IARC), Dietary Exposure Assessment Group (DEX), 150 Cours Albert Thomas, 69372 Lyon, France

**Keywords:** Mediterranean diet score, Blood cholesterol, Cardiovascular disease, Dietary pattern analysis

## Abstract

**Background:**

Blood lipids are cardiovascular health indicators. High LDL cholesterol values and/or high total cholesterol (TC)/HDL cholesterol ratios are positively related with cardiovascular mortality. Evidence suggests that a Mediterranean diet can reduce the incidence of cardiovascular diseases. Adherence to the Mediterranean diet is often measured by the Mediterranean Diet Score (MDS). However, the association between the Mediterranean diet and blood lipid profiles seems still inconclusive. The aim of this study was to investigate the relationship between the MDS, its different components and blood lipid profiles.

**Methods:**

A sample of 506 women and 707 men (aged 18–75 years) was recruited. Three-day diet records were used to calculate the MDS. Blood samples were analyzed for serum TC, LDL and HDL cholesterol. ANOVA was used to analyze blood lipids across the MDS tertiles. A multivariate linear regression analysis was performed to investigate the associations between the MDS, its components and blood lipids, adjusted for several confounders. All analyses were stratified by gender.

**Results:**

Few gender-specific associations were found between the MDS, its components and blood lipids. Only in men, the total MDS was negatively related with LDL cholesterol and the ratio TC/HDL cholesterol while positively with HDL cholesterol. In women, respectively two (MUFA/SFA and cereals) and in men three (fruits & nuts, meat and alcohol) of the nine MDS components were related with blood lipids.

**Conclusions:**

Analyses investigating the relationship between the MDS, its components and blood lipid profiles indicate only limited influence of the Mediterranean diet on blood lipids. More associations were detected in men compared to women.

## Background

Relationships between dietary patterns and quality of life have been widely investigated [1]. Particularly the adherence to a Mediterranean dietary pattern is likely to be associated with positive health outcomes. Willett et al. [2] reported that, together with non-smoking and regular physical activity, over 80% of coronary heart disease (CHD), 70% of stroke and 90% of type two diabetes can be avoided by making food choices which are consistent with the traditional Mediterranean dietary pattern. This pattern is defined by a high intake of plant foods, monounsaturated fats (MUFA), fish and whole grains; moderate intake of ethanol and dairy products; and low intake of meat, refined grains and sweets [3]. The Mediterranean dietary pattern was brought to the attention by Ancel Keys, who investigated the relationship between dietary fatty acids, nutritional cholesterol intake and 25-year mortality from CHD in the Seven Countries Study [4].

The research approach of assessing single nutrients and food groups in relation to the prevalence of diseases has proven associations with health outcomes [5, 6]. Furthermore, investigators have suggested that a more holistic dietary approach on disease prevention could be recommended [7, 8]. An internationally validated scale to assess the adherence to the Mediterranean dietary pattern was developed by Trichopoulou et al. [9]. The Mediterranean Diet Score (MDS) consists of nine single components, namely the component monounsaturated/saturated fatty acids (MUFA/SFA), legumes, fruits and nuts, vegetables, meat, cereals, alcohol, dairy and fish. Sofi et al. [10] described that a two point increase of the MDS leads to a 9% reduction in cardiovascular mortality in different non-Mediterranean populations. It was hypothesized that the positive effect of MDS on cardiovascular health is mediated by blood lipids. Several reports suggested that MDS is associated with blood lipids. Pitsavos et al. [11] and Panagiotakos et al. [12] showed that the MDS was inversely associated with LDL cholesterol. Carter et al. [13] reported that the ratio total cholesterol (TC)/HDL cholesterol decreased with an increasing MDS and that HDL cholesterol increased with increasing MDS tertiles. Using an alternative calculation of the MDS by examining questions from an existing life-style questionnaire measuring the relevance to Mediterranean diet components and the adherence to Mediterranean diet patterns based on previous studies, lower LDL cholesterol and higher HDL cholesterol levels were observed in men with a higher modified MDS (mMDS) [14]. In contrast, de Lorgeril et al. [15] confirmed the cardio-protective effect of the Mediterranean dietary pattern in the Lyon Diet Heart Study, but showed no differences in blood lipids between the control group (with a classic diet low in SFA) and the experimental group (with a Mediterranean type of diet). The equivocal results regarding the association between the MDS and blood lipids may be due to the influence of several confounding factors, which could have been inconsistently taken into account. Due to the menopausal status and the use of hormone replacement therapy there are gender-specific differences in blood lipids, with women having higher levels of TC and HDL cholesterol, while men have higher LDL cholesterol levels. HDL cholesterol and LDL cholesterol are also influenced by age [16]. Gostynski et al. [17] found that hypercholesterolemia increased with age. There is also a positive relation between hypercholesterolemia and Body Mass Index (BMI). The physical activity level (PAL) mostly raises HDL cholesterol, but findings for TC and LDL cholesterol are less consistent [18]. In some studies, smoking is positively related with CHD, but the suggestion that smoking modifies the association between blood lipids and cardiovascular risk is uncertain [19].

Despite the cardio-protective effect, the relation between a Mediterranean dietary pattern, its components and blood lipids remains debated. Therefore, it might be interesting to investigate this in a Flemish population, with a wide variability in food habits and food scores [20–22] since sufficient variability is required to detect possible associations. The aim of this study was to investigate the association between the MDS, used as an indicator for the adherence to a Mediterranean dietary pattern, and blood lipids. This paper explored the relationship between the MDS, its components and blood lipids taking into account several potential confounding factors such as age, PAL, BMI, energy intake and smoking. A better blood lipid profile with an increasing MDS was hypothesized because of the high vegetable and the low SFA character of the Mediterranean dietary pattern.

## Methods

### Subjects

Data were collected by the Flemish Policy Research Centre Sport, Physical Activity and Health [23]. One of the aims of this Research Centre was to investigate the relationship between nutritional habits, physical health, mental health and physical fitness among an adult population. For this purpose, 46 Flemish municipalities were selected by clustered random sampling. Within these municipalities, a random sample of men and women between 18 and 75 years of age was selected to participate. A sample size analysis was performed with alpha = 0.05, power = 0.80 and an effect size of 5 mg/dl for TC between low and high MDS. The estimated sample size was 1132. More participants were recruited to account for possible drop-outs and invalid measures. Of the 1511 participants, 1213 completed all tests and measurements. Results are based on data of 506 women and 707 men. Although small differences with the general Flemish population were observed, the sample of the current study can be considered as sufficiently representative for geographic distribution, age, gender and educational level. Subjects were asked to visit the test laboratory to provide a fasting blood sample, to have anthropometric measurements taken and to complete questionnaires. A three-day diet record was sent about two weeks before their visit to the laboratory, and subjects were requested to bring their completed record on the day of their appointment. All participants signed an informed consent form and received information about the tests and measurements. The study was approved by the ethical and medical committee of the Katholieke Universiteit Leuven.

### Measurements

#### Dietary assessment

Participants completed a three-day diet record, in which they noted all foods and drinks during two weekdays and one weekend day [24]. The participants were instructed to weigh the amount of foods and drinks consumed. If weighing was not possible, the participants were instructed to estimate the amount of the foods and drinks they consumed by using standard household measures (e.g. a spoon, glass, cup, etc.). All information about the diet record was included in the three-day record booklet. Diet records were analyzed using Becel Nutrition software (Unilever Co.; Rotterdam, The Netherlands). Total energy intake (in kcal/day), consumption of food groups (in gram/day), macronutrients (in gram/day) and micronutrients (in mg/day or μg/day) were calculated. A nine-point score of the MDS was calculated using gender-specific median of intakes [9]. For beneficial components (as assigned by the MDS) such as vegetables, legumes, fruits and nuts, cereal and fish, a consumption below the median was assigned a value of 0, and a consumption above the median was assigned a value of 1. For the detrimental components (as assigned by the MDS) meat, poultry and dairy products, a consumption below the median was assigned a value of 1, and a consumption above the median received a value of 0. Men with a consumption of ethanol between 10 and 50 g per day and women with a consumption of ethanol between 5 and 25 g per day had a value of 1, other consumption patterns for ethanol received a value of 0. For fat intake the ratio MUFA/SFA was determined. A value above the median was rated 1, below the median 0. Thus, the total MDS is calculated as the sum of the above mentioned component scores.

### Blood samples

Participants were instructed to fast from 11:00 p.m. the evening before their visit to the laboratory. A fasting blood sample was taken by a nurse or physician from an antecubital vein in supine position. A tube of 10 ml Venoject was used to determine TC and HDL cholesterol. After standing for 20 to 30 minutes at room temperature, samples were placed in the fridge (4°C) vertically and were analyzed by the laboratory on the same day. TC and HDL cholesterol was analyzed using the homogeneous polyanion/cholesterol esterase/oxidase enzymatic method using an Olympus AU5400 analyzer (Olympus Diagnostica, Hamburg, Germany). LDL cholesterol was calculated using the following formula: LDL cholesterol = TC – HDL cholesterol – Triglycerides/5 [25].

### Anthropometric measurements

Anthropometric measurements were performed by trained staff using standardized techniques and equipment according to the International Society for the Advancement of Kinanthropometry [26]. Participants were measured barefoot and in minimal clothing. Body weight was recorded to the nearest 0.1 kg with a digital balance (Seca 841, Seca GmbH, Hamburg, Germany) and body height with a Holtain stadiometer (Holtain, Crymych, UK) to the nearest 0.1 cm. BMI was calculated using the following formula: BMI = body weight (kg)/(height (m))^2^.

### Physical activity level (PAL)

The PAL was estimated using the Flemish Physical Activity Questionnaire (FPACQ). The FPACQ was found to be a valid and reliable questionnaire for the different components of physical activity during a normal week in adults [27]. The PAL is an indicator of the general activity level expressed in relation to the basal metabolism. A PAL between 1.40 and 1.69 is defined as sedentary, between 1.70 and 1.99 as moderately active and from 2.00 onwards as vigorously active [28].

### Smoking behavior

Smoking behavior was assessed using the WHO Monica Smoking Questionnaire enabling dichotomization in actual smokers and non-smokers [29].

### Statistical analysis

SPSS 21.0 (SPSS Inc. Chicago, IL) statistics software was used for data analysis. Descriptive statistics, such as mean and standard deviation were calculated for characterization of the participants. The distribution of age, BMI, total energy intake, PAL and blood lipids across the tertiles of the MDS was tested using ANOVA (p for trend). The association between the MDS and actual smokers was determined by a chi-square test. Associations between the MDS, its components and blood lipids were tested using multivariate linear regression (forced entry method) with blood lipids as a continuous dependent variable, adjusted for age, BMI, total energy intake and PAL as continuous variables and smoking as a dummy variable. All analyses were stratified by gender. A two-sided 0.05 level of significance was defined. Normality of the data was checked visually and with Kolmogorov-Smirnov test. Data were mostly normally distributed. Non-normally distributed data were tested with parametric and non-parametric tests, this did not influence the nature of the relationships. For clarity and simplicity, we presented only the results of the parametric tests.

## Results

Sample characteristics are given in Table 1. Of all the participants, 15% were actual smokers. The mean (SD) age was 44.7 (10.3) years for women and 46.5 (11.4) years for men. Women and men had a mean (SD) BMI of 24.2 (3.9) kg/m^2^ and 25.6 (3.1) kg/m^2^ respectively. Mean (SD) daily energy intake was 1976 (533) kcal for women and 2565 (749) kcal for men, and PAL was 1.7 (0.2) for women and 1.8 (0.3) for men. TC was 205 (37) mg/dl for women and 208 (38) mg/dl for men and ratio TC/HDL cholesterol was 3.2 (0.9) and 3.9 (1.0) respectively. The mean (SD) total MDS was 4.6 (1.8) for women and 4.7 (1.7) for men.

**Table 1:** **Characteristics of the participants**

	Women (n = 506)		Men (n = 707)	
	Mean	SD	Mean	SD
Age (years)	44.7	10.3	46.5	11.4
BMI (kg/m^2^)	24.2	3.9	25.6	3.1
Total energy intake (kcal)	1976	533	2565	749
Physical activity level	1.7	0.2	1.8	0.3
Total cholesterol (mg/dl)	205	37	208	38
LDL cholesterol (mg/dl)	118	33	130	35
HDL cholesterol (mg/dl)	67	15	55	12
Total/HDL cholesterol	3.2	0.9	3.9	1.0
MDS total	4.6	1.8	4.7	1.7
	n	%	n	%
Actual smokers	78	15	103	15

Table 2 illustrates the distribution of age, BMI, energy intake, PAL, actual smokers and blood lipids as a function of the MDS tertiles, stratified by gender. For both genders the mean age increased as a function of the MDS tertiles (p < 0.001). There were no statistically significant differences in BMI, total energy intake, PAL and actual smokers between the MDS tertiles. In men, there was a statistically significant difference in smoking (p = 0.046), with the lowest prevalence in the highest MDS tertile. HDL cholesterol increased (p = 0.001) and ratio TC/HDL cholesterol decreased (p = 0.005) with increasing tertile in men.

**Table 2:** **Age, BMI, total energy intake, PAL and actual smokers as a function of MDS tertiles**

Women	Mediterranean diet score						
	0 to 3 points		4 to 6 points		7 to 9 points		P for trend
	Mean	SD	Mean	SD	Mean	SD	
Age (years)	42.0	9.9	45.1	10.0	47.7	11.0	<0.001
BMI (kg/m^2^)	24.2	3.8	24.2	3.9	24.4	3.8	0.676
Total energy intake (kcal)	1883	463	2007	570	2005	481	0.105
Physical activity level	1.7	0.1	1.7	0.2	1.7	0.1	0.681
	n	%	n	%	n	%	P chi square
Actual smokers	26	33	45	58	7	9	0.064
Total cholesterol (mg/dl)	204	35	205	37	206	42	0.758
LDL cholesterol (mg/dl)	120	31	117	33	121	37	0.920
HDL cholesterol (mg/dl)	66	13	68	16	66	15	0.844
Total/HDL cholesterol	3.2	0.7	3.1	0.9	3.2	0.9	0.733
**Men**							
	**Mean**	**SD**	**Mean**	**SD**	**Mean**	**SD**	
Age (years)	43.4	11.8	46.9	10.9	49.7	11.6	<0.001
BMI (kg/m^2^)	25.2	3.2	25.8	3.1	25.4	3.0	0.057
Total energy intake (kcal)	2462	774	2593	771	2617	596	0.093
Physical activity level	1.8	0.2	1.8	0.3	1.8	0.3	0.222
	n	%	n	%	n	%	P chi square
Actual smokers	34	33	57	55	12	12	0.046
Total cholesterol (mg/dl)	210	42	207	37	208	34	0.599
LDL cholesterol (mg/dl)	133	39	129	34	127	31	0.161
HDL cholesterol (mg/dl)	53	11	55	12	58	13	0.001
Total/HDL cholesterol	4.1	1.1	3.9	1.0	3.7	0.9	0.005

The results of the association between the MDS, its components and blood lipids adjusted for age, BMI, energy intake, PAL and smoking behavior stratified by gender are shown in Table 3. The component MUFA/SFA was negatively related with HDL cholesterol while positively with the ratio TC/HDL cholesterol in women. The component fruits and nuts was negatively related with TC, LDL cholesterol and the ratio TC/HDL cholesterol while positively with HDL cholesterol in men. For the meat component, there was a negative relation with TC among men. The cereals component was negatively related with TC, LDL cholesterol and the ratio TC/HDL cholesterol in women. The alcohol component was positively related with TC and HDL cholesterol in men. For total MDS only a relationship was seen in men. MDS was negatively related with LDL cholesterol and the ratio TC/HDL cholesterol, while positively with HDL cholesterol.

**Table 3:** **Multivariate linear regression with blood lipids as a continuous dependent variable: associations with the MDS and components**

	Women (n = 506)				Men (n = 707)			
	Total cholesterol	LDL cholesterol	HDL cholesterol	TC/HDL cholesterol	Total cholesterol	LDL cholesterol	HDL cholesterol	TC/HDL cholesterol
Component MUFA/SFA	-1.95 (-8.05; 4.15)	2.80 (-3.24; 7.80)	-3.54 (-6.10; -0.97)*	0.14 (0.01; 0.28)*	2.81 (-2.67; 8.28)	1.40 (-3.60; 6.40)	0.65 (-1.01; 2.31)	-0.01 (-0.15; 0.13)
Component legumes	-7.90 (-17.38; 1.57)	-4.42 (-13.01; 4.17)	-2.84 (-6.85; 1.17)	-0.03 (-0.24; 0.19)	4.38 (-5.18; 13.94)	3.94 (-4.78; 12.66)	-1.21 (-4.10; 1.68)	0.16 (-0.09; 0.40)
Component fruits and nuts	-2.67 (-8.87; 3.53)	-1.33 (-6.94; 4.28)	-1.14 (-3.76; 1.48)	-0.02 (-0.16; 0.12)	-9.29 (-14.87; -3.71)*	-9.36 (-14.44; -4.23)*	1.75 (0.05; 3.44)*	-0.28 (-0.42; -0.13)*
Component vegetables	-1.49 (-7.81; 4.84)	-0.57 (-6.29; 5.16)	0.17 (-2.84; 2.51)	-0.02 (-0.16; 0.12)	-0.37 (-6.24; 5.49)	0.24 (-5.12; 5.59)	-0.84 (-2.61; 0.94)	0.04 (-0.11; 0.20)
Component meat	1.45 (-5.01; 7.92)	0.65 (-6.49; 5.20)	1.27 (-1.46; 4.00)	0.02 (-0.13; 0.16)	-5.99 (-11.76; -0.22)*	-4.56 (-9.83; 0.71)	-0.93 (-0.68; 0.82)	-0.04 (-0.19; 0.11)
Component cereals	-8.78 (-15.64; -1.92)*	-10.88 (-17.05; -4.71)*	1.04 (-1.88; 3.96)	-0.21 (-0.36; -0.05)*	-1.51 (-7.71; 4.70)	-0.90 (-6.56; 4.77)	-1.57 (-3.44; 0.31)	0.09 (-0.06; 0.26)
Component alcohol	0.11 (-0.63; 6.45)	-2.34 (-8.07; 3.39)	1.87 (-80; 4.55)	-0.10 (-0.24; 0.04)	6.88 (1.42: 12.33)*	2.26 (-2.74; 7.26)	3.29 (1.66; 4.93)*	-0.11 (-0.26; 0.03)
Component dairy	-2.67 (-8.84; 3.35)	-1.79 (-7.36; 3.81)	0.59 (-2.02; 3.20)	-0.07 (-0.20; 0.07)	2.51 (-3.18; 8.19)	0.67 (-4.53; 5.86)	1.58 (-0.14; 3.29)	-0.05 (-0.19; 0.10)
Component fish	2.27 (-3.81; 8.36)	0.52 (-4.99; 6.03)	1.71 (-0.86; 4.28)	-0.01 (-0.15; 0.12)	-0.99 (-6.48; 4.48)	-1.40 (-6.40; 3.60)	0.47 (-1.19; 2.13)	-0.08 (-0.22; 0.07)
Mediterranean diet score	-1.36 (-3.08; 0.34)	-1.19 (-2.74; 0.36)	-0.07 (-0.79; 0.65)	-0.02 (-0.06; 0.02)	-1.01 (-2.75; 0.55)	-1.66 (-3.17; -0.15)*	0.54 (0.05; 1.05)*	-0.06 (-0.10; -0.02)*

*p < 0.05; adjusted for age (in years), Body Mass Index (in kg/m^2^), total energy intake (in kcal), physical activity level (in PAL) and smoking (yes versus no).

## Discussion

The aim of the present study was to investigate the association between the MDS, its components and blood lipids, taking into account potential confounding factors such as age, BMI, PAL, energy intake and smoking. The results showed that there are only limited gender-specific relationships between total MDS or its components and blood lipids. A better blood lipid profile with an increasing MDS was hypothesized since the Mediterranean dietary pattern is characterized by a high consumption of vegetable food (including vegetables, pulses and whole cereals), a regular use of olive oil and low SFA. These factors are related with lower TC levels, which are considered as health protective [4, 30]. In our study, only weak associations with blood lipids were found in the non-adjusted and in the adjusted analysis. The results are more in accordance with the formulated hypothesis in men compared to women. The negative relation between MDS and LDL cholesterol in the adjusted analysis corroborate the findings of Pitsavos et al. [11], Panagiotakos et al. [12] and Fitó et al. [31], but in men only. Carter et al. [13] reported an increase in HDL cholesterol and a decrease in TC/HDL cholesterol with an increasing MDS. In the present study the same association was found, but only in men. Pitsavos et al. [11] and Carter et al. [13] found the same results in men and women. Panagiotakos et al. [12] adjusted for gender and Yang et al. [14] only included men. Different relations between the MDS and blood lipid profiles found for men compared to women can be explained by the fact that some physiologic and metabolic factors may induce other responses in women compared with men, such as menopausal status and the use of hormone replacement therapy [16]. Genetic differences may also induce different responses in the blood lipid profile. There are some effects of estrogen that may predict benefits [32].

The weak relations found in our study are in line with the results from the Lyon Diet Heart study, in which was found that a Mediterranean diet does not qualitatively alter the reported relationships between the risk factors of CHD, such as blood lipids, and the recurrence rate of CHD [33]. Moreover, the association between CHD and the dietary intake of polyunsaturated fatty acids (PUFA) and SFA was contested by the meta-analysis of Chowdhury et al. [34], concluding that there is no clear evidence for the present cardiovascular guidelines encouraging low consumption of SFA and high consumption of PUFA.

As a consequence, uncertainties remain about the cardio-protective mechanism of the Mediterranean diet. According to de Lorgeril et al. [35], other Mediterranean lifestyle factors such as the absence of stress and pollution, the economic situation and the presence of health systems and extensive welfare are postulated as possible explanations for the protective effect. However, also in non-Mediterranean populations health effects can be obtained by adherence to a Mediterranean diet [36]. The reduction in CHD prevalence with comparable lipid and lipoprotein risk factors points to other important risk factor modifications influencing the development of CHD [37]. Oxidative stress due to the antioxidant capacity of the diet, decreased inflammation and improved parameters of endothelial function are possible mechanisms linking the Mediterranean diet to reduced cardiovascular risk, whilst alpha-linolenic acid can contribute to the cardio-protective abilities [38, 39]. The role of PUFA, nitrite and polyphenols can also lower the cardiovascular recurrence rate [40]. Another explanation for the lack of association between the Mediterranean dietary pattern and the blood lipid profile may be due to the influence of confounding factors. Adherence to a Mediterranean diet combined with other healthy lifestyle and genetic factors [32] may have an influence on the blood lipid profile. The latter factors are understudied in most reports, whilst they may help to explain the blurred relations between the MDS and blood lipids.

Comparing our results with those from other studies is complex, because little attention is given to confounders in most reports. Possible confounding factors are generally not clearly reported or controlled for in different ways, for example as a dichotomous variable, in more categories or in tertiles. Pitsavos et al. [11] and Yang et al. [14] reported the relation between the MDS and blood lipids in adjusted and non-adjusted analyses. Reductions in LDL cholesterol with an increasing MDS were found irrespective of various confounding factors [11]. In the study of Yang et al. [14] only LDL cholesterol varied whether the analysis was adjusted or not. For HDL cholesterol and the ratio TC/HDL cholesterol, similar results were found for non-adjusted and adjusted analysis. Yang et al. [14] reported using an adjusted analysis lower LDL cholesterol with a higher mMDS as in the adjusted analysis of the present study and higher HDL cholesterol with a higher mMDS as in both the adjusted and non-adjusted analysis of the present study.

There are some limitations to the present study. The study has a cross-sectional design and can therefore not predict causality. Another limitation of the study is that dietary records and physical activity questionnaires are susceptible to reporting bias, and a healthy volunteer effect can be suspected. Adjustment for known influencing factors was done, but factors such as pollution, stress, economic factors and genetic factors were unknown. Literature indicates that blood lipids levels seem to be higher in postmenopausal women compared to premenopausal women [41]. Information concerning menopausal status in women was not available. Hence, correction for this potential confounder was impossible. Equally, a review showed that blood lipids improved when people stopped smoking [42]. In the present study smoking status was used as a possible confounding factor. Based on the questionnaire it was not possible to divide the participants into three smoking categories (former smoker, current smoker or non-smoker). Besides the high power and the integration of confounding factors, an important strength of this study is the debated research topic. The relationship between the MDS and blood lipids is insufficiently investigated whereas underlying mechanisms and confounding factors remain unclear. Hence, reports of non-significant results are likely to remain unpublished because of publication bias [43]. Prospective research is needed to learn more about the associations between the MDS and blood lipids, potentially in combination with a cardiovascular endpoint.

Although the hypothesized relation between MDS, its components and blood lipids was not confirmed, previous studies have shown that adherence to the Mediterranean diet can be health protective [10]. Literature shows that an increment in HDL cholesterol of 1 mg/dl is estimated to decrease the risk of CHD by 2% to 3% [44]. According to earlier published papers, both the MDS and blood lipid profile seem to be separately associated with CHD. In the present study, there were only few associations between MDS, its components and blood lipids, even after adjustment for potential confounding factors.

## Conclusions

The health effect of the MDS is only slightly mediated by blood lipids, and correcting for potential confounding factors has a limited influence on the associations between the MDS and blood lipids. The analysis of the different components of the MDS does not give a further explanation for the relationships. The associations are more pronounced in men compared to women. Further research is necessary to elucidate the relationship between adherence to a Mediterranean diet and blood lipids, with special attention to confounding factors such as lifestyle and genetic factors.

## References

[1] Henríquez Sánchez P, Ruano C, De Irala J, Ruiz-Canela M, Martínez-González MA, Sánchez-Villegas A (2012). Adherence to the Mediterranean diet and quality of life in the SUN Project. Eur J Clin Nutr.

[2] Willett WC (2006). The Mediterranean diet: science and practice. Public Health Nutr.

[3] Willett WC, Sacks F, Trichopoulou A, Drescher G, Ferro-Luzzi A, Helsing E, Trichopoulos D (1995). Mediterranean diet pyramid: a cultural model for healthy eating. Am J Clin Nutr.

[4] Keys A, Menotti A, Aravanis C, Blackburn H, Djordevic BS, Buzina R, Dontas AS, Fidanza F, Karvonen MJ, Kimura N (1984). The seven countries study: 2,289 deaths in 15 years. Prev Med.

[5] Kromhout D, Bosschieter EB, De Lezenne Coulander C (1985). The inverse relation between fish consumption and 20-year mortality from coronary heart disease. N Engl J Med.

[6] Genkinger JM, Koushik A (2007). Meat consumption and cancer risk. PLoS Med.

[7] Jacques PF, Tucker KL (2001). Are dietary patterns useful for understanding the role of diet in chronic disease?. Am J Clin Nutr.

[8] Lagiou P, Trichopoulos D, Sandin S, Lagiou A, Mucci L, Wolk A, Weiderpass E, Adami HO (2006). Mediterranean dietary pattern and mortality among young women: a cohort study in Sweden. Br J Nutr.

[9] Trichopoulou A, Costacou T, Bamia C, Trichopoulos D (2003). Adherence to a Mediterranean diet and survival in a Greek population. N Engl J Med.

[10] Sofi F, Macchi C, Abbate R, Gensini GF, Casini A (2013). Mediterranean diet and health status: an updated meta-analysis and a proposal for a literature-based adherence score. Public Health Nutr.

[11] Pitsavos C, Panagiotakos DB, Tzima N, Chrysohoou C, Economou M, Zampelas A, Stefanadis C (2005). Adherence to the Mediterranean diet is associated with total antioxidant capacity in healthy adults: the ATTICA study. Am J Clin Nutr.

[12] Panagiotakos D, Pitsavos C, Stefanadis C (2006). Dietary patterns: a Mediterranean diet score and its relation to clinical and biological markers of cardiovascular disease risk. Nutr Metab Cardiovasc Dis.

[13] Carter SJ, Roberts MB, Salter J, Eaton CB (2010). Relationship between Mediterranean Diet Score and atherothrombotic risk: findings from the Third National Health and Nutrition Examination Survey (NHANES III), 1988–1994. Atherosclerosis.

[14] Yang J, Farioli A, Korre M, Kales SN (2014). Modified mediterranean diet score and cardiovascular risk in a North American working population. PLoS One.

[15] De Lorgeril M, Salen P (2005). Dietary prevention of coronary heart disease: the Lyon diet heart study and after. World Rev Nutr Diet.

[16] Monda KL, Ballantyne CM, North KE (2009). Longitudinal impact of physical activity on lipid profiles in middle-aged adults: the Atherosclerosis Risk in Communities Study. J Lipid Res.

[17] Gostynski M, Gutzwiller F, Kuulasmaa K, Döring A, Ferrario M, Grafnetter D, Pajak A, Project WM (2004). Analysis of the relationship between total cholesterol, age, body mass index among males and females in the WHO MONICA Project. Int J Obes Relat Metab Disord.

[18] Ki M, Pouliou T, Li L, Power C (2011). Physical (in)activity over 20 y in adulthood: associations with adult lipid levels in the 1958 British birth cohort. Atherosclerosis.

[19] Huxley R, Nakamura K, Woodward M (2010). Modification of the effect of lipids on the risk of cardiovascular diseases by cigarette smoking. Clinical Lipidology.

[20] Temme E, Huybrechts I, Vandevijvere S, De Henauw S, Leveque A, Kornitzer M, De Backer G, Van Oyen H (2010). Energy and macronutrient intakes in Belgium: results from the first National Food Consumption Survey. Br J Nutr.

[21] Roos G, Johansson L, Kasmel A, Klumbiene J, Prattala R (2001). Disparities in vegetable and fruit consumption: European cases from the north to the south. Public Health Nutr.

[22] Mullie P, Guelinckx I, Clarys P, Degrave E, Hulens M, Vansant G (2009). Cultural, socioeconomic and nutritional determinants of functional food consumption patterns. Eur J Clin Nutr.

[23] Duvigneaud N, Wijndaele K, Matton L, Deriemaeker P, Philippaerts R, Lefevre J, Thomis M, Duquet W (2007). Socio-economic and lifestyle factors associated with overweight in Flemish adult men and women. BMC Public Health.

[24] Deriemaeker P, Aerenhouts D, Hebbelinck M, Clarys P (2006). Validation of a 3-day diet diary: comparison with a 7-day diet diary and a FFQ. Med Sci Sports Exerc.

[25] Friedewald WT, Levy RI, Fredrickson DS (1972). Estimation of the concentration of low-density lipoprotein cholesterol in plasma, without use of the preparative ultracentrifuge. Clin Chem.

[26] Stewart A, Marfell-Jones M, Olds T, De Ridder H (2001). International Society for the Advancement of Kinanthropometry: International Standards for Anthropometric Assessment.

[27] Matton L, Wijndaele K, Duvigneaud N, Duquet W, Philippaerts R, Thomis M, Lefevre J (2007). Reliability and validity of the flemish physical activity computerized questionnaire in adults. Res Q Exerc Sport.

[28] World Health Organisation (2005). Human energy requirements: report of a joint FAO/ WHO/UNU Expert Consultation. Food Nutr Bull.

[29] ᅟ (1988). The Monica Project of the “Brianza Area”: distribution of coronary risk factors. G Ital Cardiol.

[30] Kromhout D (1999). Serum cholesterol in cross-cultural perspective: the seven countries study. Acta Cardiol.

[31] Fitó M, Guxens M, Corella D, Sáez G, Estruch R, De la Torre R, Francés F, Cabezas C, López-Sabater Me C, Marrugat J, García-Arellano A, Arós F, Ruiz-Gutierrez V, Ros E, Salas-Salvadó J, Fiol M, Solá R, Covas MI (2007). Effect of a traditional Mediterranean diet on lipoprotein oxidation: a randomized controlled trial. Arch Intern Med.

[32] Rossouw JE (2002). Hormones, genetic factors, and gender differences in cardiovascular disease. Cardiovasc Res.

[33] De Lorgeril M, Salen P, Martin JL, Monjaud I, Delaye J, Mamelle N (1999). Mediterranean diet, traditional risk factors, and the rate of cardiovascular complications after myocardial infarction: final report of the Lyon Diet Heart Study. Circulation.

[34] Chowdhury R, Warnakula S, Kunutsor S, Crowe F, Ward HA, Johnson L, Franco OH, Butterworth AS, Forouhi NG, Thompson SG, Khaw KT, Mozaffarian D, Danesh J, Di Angelantonio E (2014). Association of dietary, circulating, and supplement fatty acids with coronary risk: a systematic review and meta-analysis. Ann Intern Med.

[35] De Lorgeril M, Salen P, Paillard F, Laporte F, Boucher F, De Leiris J (2002). Mediterranean diet and the French paradox: two distinct biogeographic concepts for one consolidated scientific theory on the role of nutrition in coronary heart disease. Cardiovasc Res.

[36] De Lorgeril M (2013). Mediterranean diet and cardiovascular disease: historical perspective and latest evidence. Curr Atheroscler Rep.

[37] Kris-Etherton P, Eckel RH, Howard BV, St Jeor S, Bazzarre TL, Association NCPSCaCSCotAH (2001). AHA science advisory: Lyon Diet Heart Study: benefits of a Mediterranean-style, National Cholesterol Education Program/American Heart Association Step I Dietary Pattern on Cardiovascular Disease. Circulation.

[38] Dai J, Jones D, Goldberg J, Ziegler T, Bostick R, Wilson P, Manatunga A, Shallenberger L, Jones L, Vaccarino V (2008). Association between adherence to the Mediterranean diet and oxidative stress. Am J Clin Nutr.

[39] Schwingshackl L, Hoffmann G (2014). Mediterranean dietary pattern, inflammation and endothelial function: a systematic review and meta-analysis of intervention trials. Nutr Metab Cardiovasc Dis.

[40] Nadtochiy SM, Redman EK (2011). Mediterranean diet and cardioprotection: the role of nitrite, polyunsaturated fatty acids, and polyphenols. Nutrition.

[41] Torng PL, Su TC, Sung FC, Chien KL, Huang SC, Chow SN, Lee YT (2002). Effects of menopause on intraindividual changes in serum lipids, blood pressure, and body weight–the Chin-Shan Community Cardiovascular Cohort study. Atherosclerosis.

[42] Forey BA, Fry JS, Lee PN, Thornton AJ, Coombs KJ (2013). The effect of quitting smoking on HDL-cholesterol - a review based on within-subject changes. Biomark Res.

[43] Von Elm E, Röllin A, Blümle A, Huwiler K, Witschi M, Egger M (2008). Publication and non-publication of clinical trials: longitudinal study of applications submitted to a research ethics committee. Swiss Med Wkly.

[44] Gordon DJ, Probstfield JL, Garrison RJ, Neaton JD, Castelli WP, Knoke JD, Jacobs DR, Bangdiwala S, Tyroler HA (1989). High-density lipoprotein cholesterol and cardiovascular disease: four prospective American studies. Circulation.

